# Advances in non-invasive biosensing measures to monitor wound healing progression

**DOI:** 10.3389/fbioe.2022.952198

**Published:** 2022-09-23

**Authors:** Walker D. Short, Oluyinka O. Olutoye, Benjamin W. Padon, Umang M. Parikh, Daniel Colchado, Hima Vangapandu, Shayan Shams, Taiyun Chi, Jangwook P. Jung, Swathi Balaji

**Affiliations:** ^1^ Laboratory for Regenerative Tissue Repair, Division of Pediatric Surgery, Department of Surgery, Texas Children’s Hospital and Baylor College of Medicine, Houston, TX, United States; ^2^ Department of Applied Data Science, San Jose State University, San Jose, CA, United States; ^3^ School of Biomedical Informatics, University of Texas Health Science Center, Houston, TX, United States; ^4^ Department of Electrical and Computer Engineering, Rice University, Houston, TX, United States; ^5^ Department of Biological Engineering, Louisiana State University, Baton Rouge, LA, United States

**Keywords:** wound healing, extracellular matrix (ECM), biofilm, biosensor, machine learning, impaired wound healing, perfusion, bioelectronics

## Abstract

Impaired wound healing is a significant financial and medical burden. The synthesis and deposition of extracellular matrix (ECM) in a new wound is a dynamic process that is constantly changing and adapting to the biochemical and biomechanical signaling from the extracellular microenvironments of the wound. This drives either a regenerative or fibrotic and scar-forming healing outcome. Disruptions in ECM deposition, structure, and composition lead to impaired healing in diseased states, such as in diabetes. Valid measures of the principal determinants of successful ECM deposition and wound healing include lack of bacterial contamination, good tissue perfusion, and reduced mechanical injury and strain. These measures are used by wound-care providers to intervene upon the healing wound to steer healing toward a more functional phenotype with improved structural integrity and healing outcomes and to prevent adverse wound developments. In this review, we discuss bioengineering advances in 1) non-invasive detection of biologic and physiologic factors of the healing wound, 2) visualizing and modeling the ECM, and 3) computational tools that efficiently evaluate the complex data acquired from the wounds based on basic science, preclinical, translational and clinical studies, that would allow us to prognosticate healing outcomes and intervene effectively. We focus on bioelectronics and biologic interfaces of the sensors and actuators for real time biosensing and actuation of the tissues. We also discuss high-resolution, advanced imaging techniques, which go beyond traditional confocal and fluorescence microscopy to visualize microscopic details of the composition of the wound matrix, linearity of collagen, and live tracking of components within the wound microenvironment. Computational modeling of the wound matrix, including partial differential equation datasets as well as machine learning models that can serve as powerful tools for physicians to guide their decision-making process are discussed.

## 1 Introduction

Wound healing is a complex physiologic process dependent on many cellular and molecular factors, most often resulting in the formation of a fibrotic scar. Human wound care and treatment burden aiming to attenuate fibrosis and avoid abnormal, pathologic healing represents a substantial portion of the global medical industry. In the United States alone, close to $1.5 billion are spent managing burns and burn-related injuries, much of which is spent on secondary interventions managing the resulting scar (Sen, 2021; Singer and Clark, 1999; Armstrong et al., 2020). Additionally, over 80 million surgical incisions are created each year on top of 12 million traumatic lacerations (Schultz and Wysocki, 2009). Over 8 million patients have chronic, non-healing wounds, the management of which costs Medicare between $28 million and $96 million annually (Volk et al., 2011; Olczyk et al., 2014). Aberrant healing can also lead to hypertrophic scars and keloid formation, which affects roughly 11 million patients annually (Eming et al., 2007; Sorg et al., 2017). Moreover, wounds are not only expensive to manage, but can be life threatening: diabetic foot ulcers have a similar mortality rate to cancer (30.5% vs. 31%) (Armstrong et al., 2020). Successfully managing wound healing would alleviate a serious financial burden on the medical system as well as significantly contribute to reducing morbidity and mortality.

**FIGURE 1:**
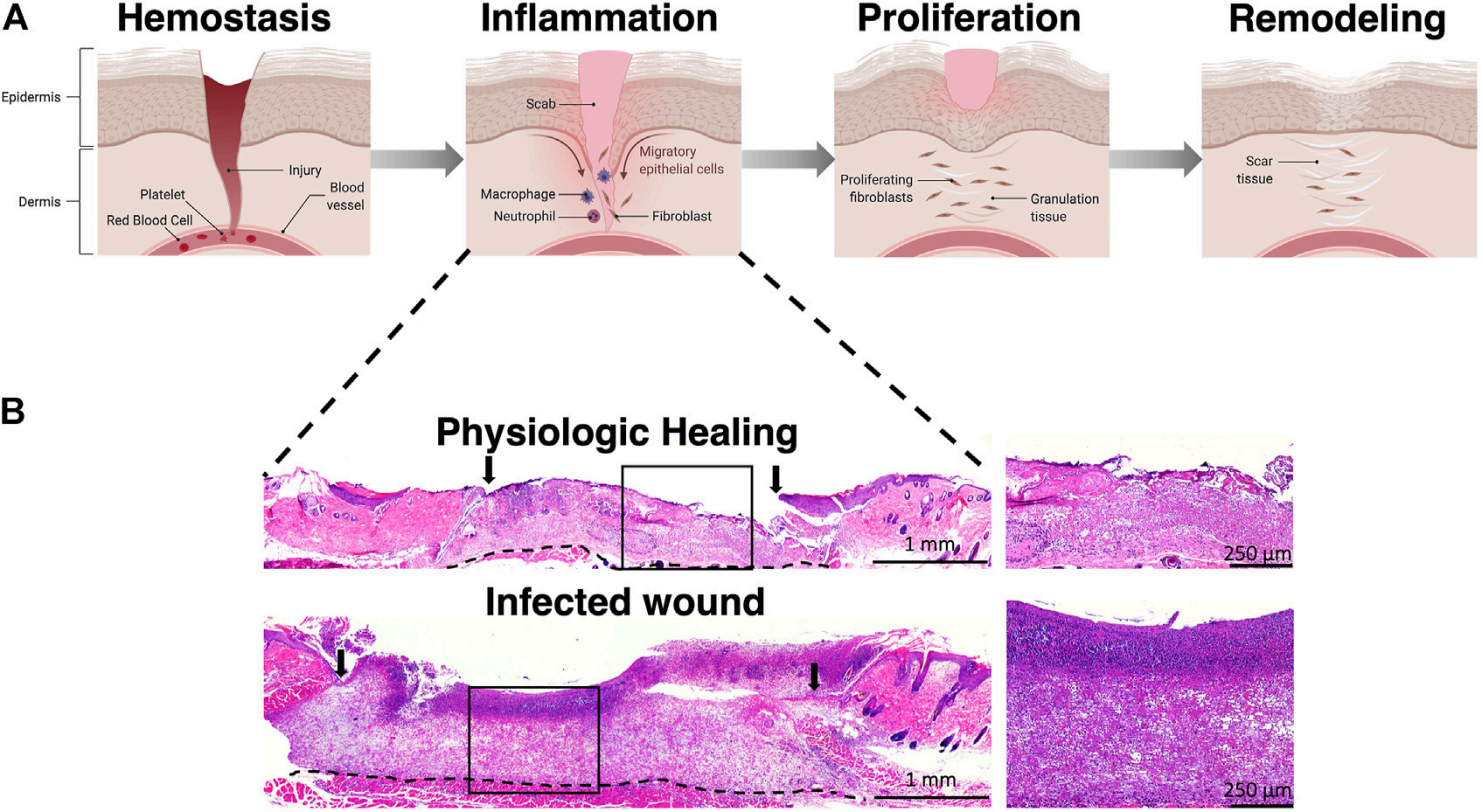
Phases of wound healing. **(A)** Wound healing progresses through four phases including hemostasis, inflammation, proliferation, and remodeling. **(B)** Representative H&E staining of wounds in the inflammation stage demonstrating poor granulation tissue formation in an infected wound. Arrows represent wound edge. Illustration created using Biorender.com.

The paragon of successful wound healing is the regenerative healing phenotype observed in midgestational fetal skin wounds. While regenerative healing mechanisms are primarily explored and modeled by researchers to alleviate the burden of scarring, inducing regenerative ECM deposition should also be targeted as an important objective in the healing of chronic wounds, which are a more pressing issue beyond cosmetic wound healing. Physiologically, wounds heal in a defined and organized manner, progressing through the overlapping phases of hemostasis, inflammation, proliferation, and remodeling (Figure 1) (Singer and Clark, 1999). In the first phase, hemostasis is achieved through vasoconstriction, activation of the coagulation cascade, and clot formation. Damaged tissue releases damage associated molecular patterns (DAMPs) that signal recruitment of neutrophils and monocytes responsible for scavenging bacteria and devitalized tissue. As the inflammation phase winds down, the proliferative phase is dominated by activated myofibroblasts and the synthesis of new extracellular matrix (ECM). The ECM not only provides structural scaffolding and integrity to the healing wound, but also simultaneously acts as a reservoir for crucial growth factors (Schultz and Wysocki, 2009), and plays an active role in regulating cell migration, differentiation, proliferation, and survival (Olczyk et al., 2014), to promote wound neovascularization and keratinocyte-mediated wound re-epithelialization. ECM synthesis and remodeling are dynamic processes. Collagen serves as the major constituent of the cutaneous ECM, with collagen type III laid down first in the healing wound which is then replaced by collagen type I during the remodeling phase, culminating in scar formation and playing a major role in the stiffness of the skin (Ben-Amor et al., 2014). Other components such as elastin, laminins, chondroitin sulphate, and proteoglycans are essential to ECM physiology, and contribute to biomechanical properties of the skin allowing for elasticity, cell signaling and sensing changes in surrounding microenvironment and shifting the structural framework of the ECM in response to micro environmental cues. Several differences among the wound ECM constituents have been shown to play a critical role in the regenerative wound healing phenotype based on the accrued literature in fetal vs. adult dermal wound healing. These differences include a higher ratio of collagen type III to collagen type I in the regenerative wound microenvironment with elevated and sustained levels of hyaluronan and an absence of elastin as compared to the fibrotic wounds. Non-invasive study of the differences in ECM patterns during the progression of healing of fetal and adult wounds will provide insights to recapitulate the regenerative phenotype.

Pathophysiologic states such as in diabetic ulcers, venous ulcers, and immunosuppression lead to a cessation of normal wound healing, leading to chronic wounds that are characterized by a prolonged inflammatory phase with dysregulated ECM, leading to dysfunctional epithelization. While normal wound healing occurs in a highly regulated fashion from “outside-in”, this coordinated process goes awry in chronic and difficult-to-heal wounds, often characterized by a large wound size and variable depths across the wound bed that are simultaneously “stuck” in different healing phases (Tredget et al., 1997; Eming et al., 2007; Gurtner et al., 2008; Ogawa, 2008; King et al., 2013; Duscher et al., 2014; Balaji et al., 2017; Sorg et al., 2017; Rodrigues et al., 2019; LeBleu and Neilson, 2020). These wounds can also be viewed in the context of disrupted dynamic reciprocity. Dynamic reciprocity (DR) is defined as ongoing, bidirectional interaction amongst cells and their surrounding microenvironment, which supports the concept that cells’ surrounding microenvironment and the cells’ function and phenotype may depend on each other. DR-driven biochemical, biophysical and cellular responses to injury play pivotal roles in regulating wound healing responses. Concerning ECM, dynamic reciprocity deems ECM an active signaling entity rather than an inert scaffold (Bissell and Aggeler, 1987; Schultz et al., 2011). DR was reported by different groups; the interaction between endothelial cells and ECM was first described by Bornstein and others in 1982 as DR, with further seminal work reporting the interaction of ECM with cells through transmembrane receptors (Bissell et al., 1982; Bornstein et al., 1982). DR is evident in all four stages of normal wound healing, with integrins playing a key role in modulating interactions between cells and the ECM. Elevated protease levels in the chronic wounds hinder ECM—cell interactions by integrin switching or lack of integrin presentation due to destroyed/damaged attachment sites for the cells in the ECM (Larjava et al., 1993; Lafrenie and Yamada, 1996; Miyamoto et al., 1996; Lobmann et al., 2002). Therefore, the functional cells in the wound are deprived of signals required for migration, proliferation, and differentiation. Further, the pathogenesis of these wounds has been linked to persistently high proteolytic activity along with excess deposition of ECM (Buccafusco and Serra, 1985; McCarty and Percival, 2013). Impaired healing and scarring also reduce the elasticity and integrity of the remodeled tissue while impairing its function (Davey et al., 2003; Corr and Hart, 2013), with even more severe complications in patients who suffer from diseases that lead to poor wound healing and chronic wounds, such as diabetes mellitus (Weiser et al., 2018).

Current metrics for evaluating successful healing of a wound rely primarily upon measuring re-epithelialization. When a wound appears to be stalled in the progression of healing, a physician investigates the factors usually involved in hampering wound healing such as bacterial infection, inadequate perfusion, and mechanical strain on the site of injury, often requiring further imaging studies or procedures. These factors negatively impact the successful deposition and remodeling of ECM in a wound. However, the current standard of care does not evaluate the integrity and quality of the ECM of the healing wound–understandably, as non-invasive methods of evaluating the ECM are not yet commonplace, and invasive assessment by sampling tissue biopsies create additional morbidity at the site of injury. Novel techniques that allow longitudinal, non-invasive, multiparametric monitoring of the wound healing progress in real time will be a significant boon to the field of wound care.

There are many new and emerging techniques that can be utilized to further evaluate biologic factors in the healing wound which ultimately determine the structure and function of the ECM of a healed wound. Multimodal electronic biosensors that have better wound interfaces can assess the wound state in real-time with enhanced spatiotemporal resolution. Similarly, mathematical models providing a theoretical map of the ECM can be used to run simulations of wound healing end points based on given inputs, which will aid in forecasting the healing outcomes of complex wounds and allow for timely interventions. Machine learning algorithms can process complex chemical signals and ECM data inputs to determine the state of wound healing with the potential to predict optimal interventions to promote wound healing. Together, these computational models along with *in vivo* animal models will allow for the integration of physiological, molecular, mechanical, and electronic processes that increase both the number of independent signals that can be monitored simultaneously and the number of stimuli that can be administered to the repairing wound tissue.

These technologies may potentially lead to a transformative wound healing approach that can expedite recovery, eliminate incomplete healing with scarring, and reduce the risks of infection and limb amputations (Zhang et al., 2021). In addition, the ability to real-time monitor the wound status without continuous screenings by medical professionals, may result in substantially reduced treatment cost and facilitate clinicians in more precisely tracking the healing process of the patients, enabling developments of personalized and predictive care. Biosensors, which actively monitor the early wound environment for bacterial infection, perfusion, and mechanical strain, will allow wound care providers the opportunity to intervene on wounds that are predicted to fail to heal. Novel non-invasive measures of the quality of the ECM deposition in the healing wound will clue in providers as to the functional dependability of the healed wound. Machine learning and deep learning will allow extraction of the mass data provided by novel biosensing and ECM visualizing modalities predicting wound failure, and perhaps 1 day intervening, before human evaluation has even taken place.

## 2 Wound interface biosensing

At the onset of the healing process, it is essential to establish an infection free wound bed along with sufficient perfusion of the granulating tissue to allow for successful ECM deposition. Development of biosensors to detect bacterial contamination through presence of a biofilm, odor, H_2_O_2_, temperature, pH, and even direct bacterial detectors as well as perfusion via oxygen sensors and near-infrared fluorescence have led to novel methods to evaluate the progression and hinderance of wound healing. Development of technologies such as these will allow physicians, particularly vascular surgeons, to rapidly identify and intervene upon wounds that display characteristics of non-healing wounds, especially in the crucial timeframe immediately following revascularization.

### 2.1 Detection of bacterial contamination and biofilms

A constant source of consternation for clinicians, infections are especially prevalent in chronic diabetic wounds, contributing to dysregulated wound healing. Sensors can be utilized to detect markers of infection like bacterial by-products and toxins as well as enzymes secreted by neutrophils (Ma et al., 2022; Nnachi et al., 2022; Ramasamy et al., 2022). About two thirds of chronic wounds present with bacteria that tend to produce a biofilm, a matrix consisting of extracellular polymeric substances (EPS) (Zhao et al., 2013). Bacterial biofilms form a protective barrier that encourages the formation of multicellular communities through the production of a complex matrix of glycoproteins and polysaccharides and hinder healing by suppressing the effectiveness of the host immune response to infection (Volden et al., 2010). Biofilms also protect bacteria from antibiotics at the concentrations which would normally kill the bacteria. Therefore, wounds are often debrided when there is a clinical suspicion of biofilm formation. Objective methods of sensing biofilm formation would provide valuable clinical information to indicate the need for wound debridement. High-frequency acoustic microscopy is a novel technique, which, when used in conjunction with targeted ultrasound contrast agents, allows for detection and quantification of bacterial biofilms (Anastasiadis et al., 2014). Another promising method for detecting biofilms in the field of wound healing is mass spectrometry. In 2013, Hines et al. demonstrated, using a diabetic rat model, the ability of ion mobility mass spectrometry (IM-MS) to accurately characterize and model wound pathology through non-invasive collection and analysis of a small sample of wound exudate (Hines et al., 2013). Mass spectrometry has also been used to characterize and quantify the presence of bacterial biofilms, including in human dermal wounds (Charlton et al., 2000; Ashrafi et al., 2018; Achek et al., 2020). In recent years, this technology has been used for the detection of metastatic breast and thyroid cancers, and has been translated into the clinic in a handheld device, the MasSpec Pen System™, for real time determination of tumor margins during pancreatic cancer surgery (Zhang et al., 2017; King et al., 2021). This technology may offer physicians a non-invasive, real-time method for analysis of a wound and determination of healing status. While this would require studies to model the healing wound and determine the variables to assess, it is a tool that could change the way wounds are assessed in the clinic and provide a method for rapid characterization of the ECM. Other indirect measures of bacterial contamination and biofilm formation include wound pH, odor, hydrogen peroxide levels, and temperature–which will be discussed in the following sections.

#### 2.1.1 pH sensors

pH is a sensitive indicator of bacterial infections in the inflammation phase. pH of normal healing wounds is in the range of 5.5–6.5 during the wound healing. However, in non-healing infected wounds, pH tends to be above 6.5 (Schneider et al., 2007). Although pH can be slightly affected by diet and diseases, the variations of wound pH in response to the infection are more severe. Thus, monitoring pH will provide important data on possible infection in the inflammation phase. pH can be easily measured with the help of indicator dyes (Kromer et al., 2022; Srivastava et al., 2022). However, care must be taken that these dyes do not leach from the dressings. Sridhar and others have developed a sensor based on a pH sensitive hydrogel between two coils (Vijayaraghavan et al., 2009). The coils displace as the hydrogel swells with changes in pH and result in a change of inductance. This kind of electronic sensor will be ideal in the future as we move toward smart wound dressings that can detect changes in pH and deploy measures to address the perturbations. pH monitoring does present difficulties, however, as the pH of a non-infected wound tends to fluctuate as a part of the physiologic wound healing response. Thus, alterations in pH are not necessarily specific to bacterial infection (Sharpe et al., 2013). Using dual-sensors, which create composite data from multiple readouts may add to the specificity of pH sensors. For example, levels of uric acid indicate the severity of the wound and decrease as the wound heals (Fernandez et al., 2012). Sharifuzzaman et al. (2020) developed a wearable biosensor for detection of uric acid, further integrated with a temperature and pH detection system to correlate changes in temperature, pH, and uric acid levels, allowing for more precise recognition of bacterial infection. Direct bacterial sensing is a much more specific measure to determine the presence and severity of microbial contamination in a wound. Preclinical dual-sensors are in development which perform real-time monitoring of pH and bacterial cellular attachment (Sheybani and Shukla, 2017). Simultaneous measurement of pH and bacterial colony size allows for a much more specific approximation of wound infection, and such a sensor could be applied to flexible materials and integrated into functional sensors such as bandages.

#### 2.1.2 Odor sensors

Bacterial colonization can also be detected via odor sensors that detect chemicals and other byproducts secreted by bacteria. These sensors, known as “electronic noses,” can identify bacterial strains based on their characteristic odor (Wiggins et al., 1985; Pavlou et al., 2002; Allardyce et al., 2006). Detection of volatile organic compounds from the wound exudate is a complex process. For example, chronic wound lesions secrete compounds detected by GC-MS, which resolves these signals. The difficulty then lies in processing the resultant plethora of information (Thomas et al., 2010). At present, the instrumentation for resolving these peaks is quite bulky. Therefore, sensors that offer portability and real-time gas detection are needed. Low-cost alternatives to gas sensors are chemiresistors and metal oxide detectors. However, these lack sensitivity in obtaining fine distinctions amongst the volatile gases.

#### 2.1.3 Hydrogen peroxide sensors

Hydrogen peroxide (H_2_O_2_) is generated in the inflammation phase of wound healing through oxidative damage, and thus, detection of H_2_O_2_ may be helpful in assessing the state of a wound and presence of contaminants. However, commercially available, enzyme-modified electrode systems to detect H_2_O_2_ usually suffer from issues such as temperature and pH stability as well as a complicated procedures for *in vivo* applications. To address this issue, recent studies suggest using platinum as the catalyst surface for direct non-enzymatic H_2_O_2_ sensing (Miao et al., 2014). In particular, Platinum-Black (Pt-Black) microelectrodes have proven to be highly sensitive for *in vivo* H_2_O_2_ measurements, which also offers superior accuracy over a large range of H_2_O_2_ concentrations (Ben-Amor et al., 2014; Du et al., 2017; Yu et al., 2021; Xie et al., 2022). By deploying an array of Pt-Black microelectrodes, the H_2_O_2_ concentration and the resulting electrochemical currents can be readily monitored using a standard potentiostat circuit.

#### 2.1.4 Temperature sensors

Temperature is a key metric for understanding not only infection, but perfusion as well. Infection is indicated in the case of elevated temperature, yet decreased temperature can be a marker of local ischemia and biofilm formation. Many temperature sensors are made of carbon nanotubes and connected to a transponder while others are colorimetric, and detect up to ±0.5°C (Zang et al., 2012). These are low cost, yet are superseded by electronic sensors that offer better sensitivity. Nanodiamonds (ND) are another material which provide non-invasive optical imaging with excellent mechanical and optical properties (Khalid et al., 2020). Khalid et al. have proposed the integration of nanodiamonds-silk materials to develop temperature sensors. These hybrids are thermally stable and are resistant to degradation as compared to silk alone, when tested in a murine model. In addition to temperature, these dressings also detect cell turnover and antibacterial activity. These arrays will allow for a direct digital output of temperature readings that can be fed back into a controller on a smart wound.

### 2.2 Detection of tissue perfusion

Perfusion of the granulating wound bed, allowing for ideal oxygenation, influx of nutrients and growth factors, and immune function is essential for proper ECM deposition. In the case of chronic wounds, there is often suboptimal macro and microvascular perfusion to the wound bed providing insufficient oxygen supply which leads to impaired healing (Chan et al., 2011; Dargaville et al., 2013). Established methods of monitoring vascular perfusion to wounds typically involve non-invasive means such as ankle brachial index, toe pressures, laser speckle perfusion mapping, and laser Doppler ultrasound flowmetry measurements performed at discreet time points for superficial wound mapping. However, these methods can be unreliable in the setting of calcified arteries common to diabetic patients with critical limb-threatening ischemia (Ricco et al., 2017). To overcome this drawback, novel advances in non-invasive measures are being explored, such as pedal acceleration time, which involves measuring the time from the start of the systolic uprise in flow in a plantar artery to the peak of systole (Sommerset et al., 2019). While pedal acceleration time has been shown to reliably measure angiosomal perfusion to foot wounds, it is not able to directly measure the perfusion to a wound as a minimally invasive peripheral angiogram would (Sommerset et al., 2019). All of these invasive and non-invasive measures of tissue perfusion share a similar drawback; they are static measures of perfusion. Continuous measures, however, may be able to detect minute changes in oxygenation, therefore informing providers of the potential need for an intervention to improve blood flow.

Given these drawbacks, there is a clinical need for non-invasive real-time measures of tissue perfusion. A step in the right direction involves the use of fluorescence angiography to determine perfusion to wounds. Indocyanine green fluorescence angiography (ICGFA), a technique progressively being used in the surgical setting, has proven to be useful in monitoring wound perfusion as well (Whitlock et al., 2021). Its use involves injection of a non-toxic and non-radioactive dye, which may then be imaged using a laser and camera under which the dye fluoresces (Patel et al., 2018). In one clinical study, poor perfusion measures by ICGFA in patients who underwent endovascular interventions were more predictive of failure of wound healing than other non-invasive measures (Patel et al., 2018). Similarly, in another study of patients with heel ulcerations, ICGFA measures successfully identified local heel ischemia and allowed for rapid vascular interventions to improve perfusion (Marmolejo and Arnold, 2018).

Another promising non-invasive measure of angiogenesis is Cadence contrast pulse sequencing, which utilizes targeted contrast agents, such as microbubbles, toward a specific vascular marker expressed by endothelium (Stieger et al., 2008; Streeter et al., 2011). This technique has been used to quantify angiogenesis *in vivo* in a murine model of mammary carcinoma (Anderson et al., 2011). Given the ubiquity of ultrasound technology, this method of measuring angiogenesis could be applied to chronic wounds in an outpatient setting.

As the principal purpose of perfusion is oxygen delivery to the healing tissue, methods of direct oxygen detection are also useful for evaluating the healing wound. Mostafalu et al. developed a 3D-printed smart wound dressing that can sense the oxygen concentrations in a wound. The bandage, along with a flexible oxygen sensor, a microcontroller and wireless radio, are all assembled into a compact system that can provide a direct data readout as well as wirelessly transmit the oxygen concentrations as the wound heals (Allardyce et al., 2006). Researchers are also implementing oxygen-sensing nanoparticles which can be directly incorporated into dressings in contact with the wound surface. These particles are excitable by ultraviolet light and emit fluorescent wavelengths which correspond to the oxygen concentration in the wound (Tavakol et al., 2020).

### 2.3 Mechanical injury and strain

Mechanotransduction is a critical factor in wound healing and fibrosis (Wang and Thampatty, 2008). Proper coordinated functional ECM deposition and wound healing rely on an environment with minimal strain and no repeated injury. Mascharak and others recently demonstrated that the fibroblasts principally responsible for dysregulated ECM deposition are activated by canonical mechanotransduction pathways, the inhibition of which allows for coordinated ECM deposition culminating in regenerative healing (Mascharak et al., 2021). Diabetic wounds, due to the underlying sensory neuropathy present in this population, are particularly susceptible to repeated injury and strain which inhibits wound healing. Wearable sensors are in development which allow for precise and constant measurements of strain to alert patients and physicians of mechanical environments, which pose a risk to the successful healing of patients’ wounds. Mehmood et al. (2015) developed a low power flexible sensing system which obtains real time pressure, moisture, and temperature data that can be wirelessly transmitted to the patient or care provider, providing insensate diabetic patients with information they can use to modify their activity and footwear. Aiming to remove the reliance of a sensor capable of measuring at only one point of the wound, Baldoli and others have developed smart wearable textiles capable of measuring distributed pressure on the foot, which also allows for measurements of pressure at the wound-dressing interface (Baldoli et al., 2016). Such sensors as above can be integrated into the wound dressings or patient socks to help in decision-making. Diagnostic sensors may dictate the course of treatment, whereas theragnostic sensors, which detect parameters such as levels of a specific protein, are then linked to a database of information regarding the wound, thus paving the way for better and personalized therapies (Harding et al., 2007; Dargaville et al., 2013).

### 2.4 Multiparameter sensors

While it is beneficial to obtain singular measures of the healing wound in real time with sensors that measure physical attributes such as temperature, odor and pH that can indicate infection and perfusion, single point measurements may not accurately assess wound tissue biology, and it is essential to be able to assess many different characteristics of a healing wound at once (El-Ali et al., 2006; Ertl et al., 2014). To assess wound healing in a more quantitative fashion, multimodal sensors that can sample multiple points in the wound are being developed (Chi et al., 2015; Park et al., 2018a; Park et al., 2018b; Park et al., 2019) In recent years, the field of microfluidics and microanalysis has allowed for the characterization and manipulation of micro/nanometer-scale volumes of fluids. These innovations have allowed for the development of multi-parametric *in situ* biosensors that prepare, manipulate, and analyze biofluids in a compact and contained manner (Whitesides, 2006; Swieszkowski et al., 2020). The biosensing technology discussed previously, along with microfluidic technology, has been integrated into *in situ* multi-parametric cell profilers, exampled in.

**FIGURE 2:**
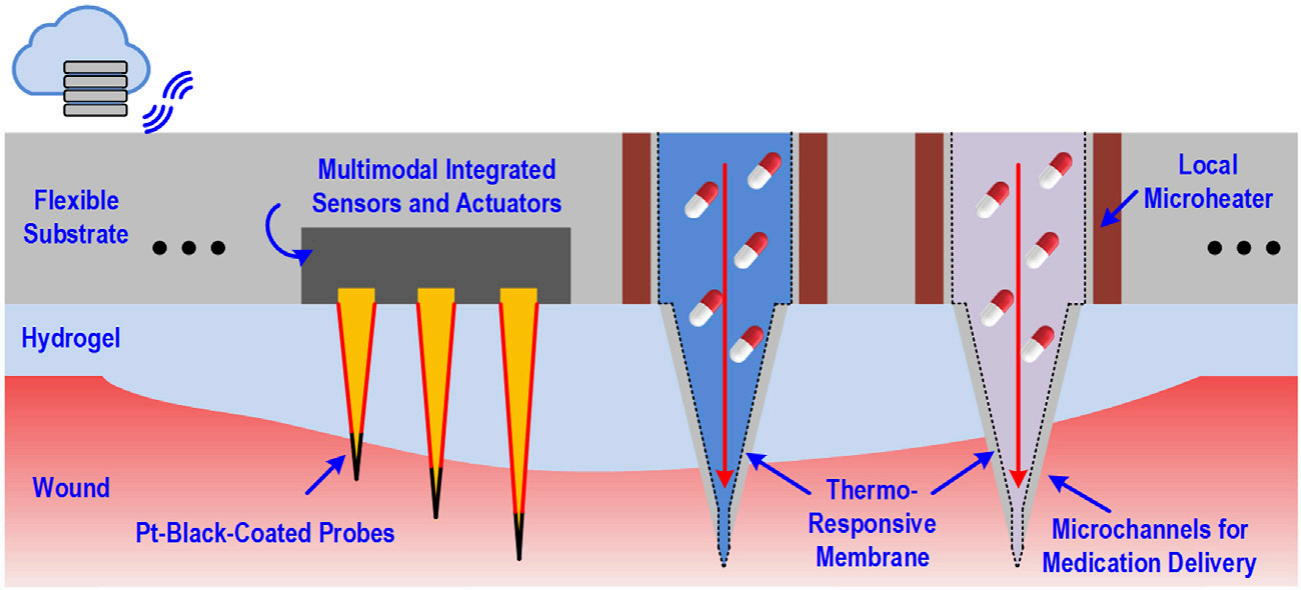
Schematic of dressing-impregnated microchip with simultaneous biosensing and medication delivery capabilities.

Park et al. This technology incorporates impedance sensing, static and dynamic optical recordings, extracellular potential recording, and biphasic current stimulation into the same 2 mm × 3 mm chip with a 1,024 pixel resolution (Park et al., 2018a). Additionally, Gao et al. developed a similar *in situ* biosensor that assess a variety of biomarkers in a venous ulcer including inflammatory mediators, bacterial load, and physiochemical parameters like temperature and pH. This device allowed real-time clinical feedback as well, incorporating a portable wireless analyzer that interfaced with the immunosensor to allow the clinician instant access to the readings taken (Gao et al., 2021). To counteract problems such as skin impedance, which makes the output signal weak, microneedle platforms can be used. Microneedle electrodes are capable of reading signals from the dermis with high sensitivity while sampling biofluids for further analysis (Chen et al., 2016; Lee et al., 2017). This technology can still be taken a step further by incorporating an on-demand drug delivery system into the wound dressing that can alter treatments in real time based on sensor readouts. A smart dressing developed by Mostafalu et al. (2018) incorporates a pH and temperature sensor along with thermosensitive drug carriers and an electronically controlled heater and onboard microcontroller to automatically release drugs onto the wound in a programmable, stimuli-responsive manner. The envisioned future generation of wound dressings will establish bidirectional communication between the wound and an electronic interface that will guide deployment of therapeutics into the healing wound in an artificial intelligence-guided closed-loop manner (Figure 2).

## 3 Visualization of the ECM

### 3.1 Advanced imaging techniques

The aforementioned sensing modalities provide data concerning predictors of successful healing, but none are objective measures of the quality of the healing wound. For this reason, we must focus on measures of the granulation tissue that provides the provisional scaffolding for the healing wound–the ECM. While invasive procedures, such as biopsy and traditional histology, have been required to evaluate the quality of wound healing and study the cell populations and demarcate the wound boundaries, they bring with them complications and confound the healing cascade by creating new injury, which impairs the assessment of the functional wound repair. Non-invasive interrogation of the functional aspects of the extracellular wound matrix is highly powerful in helping to understand the tissue repair continuum through repeated measurements of the same wound as opposed to any invasive measures. Non-invasive imaging modalities have enabled us to conduct in-depth assessment of the ECM in the wound bed and directly observe and characterize the scaffold’s mechanical structure and biomechanical properties, as well as visualize the ECM remodeling process in various pathologies. Imaging can provide a deeper understanding and greater information of the wounds non-invasively rather than single dimension diagnostics. The following sections will discuss the recent advances in ultrasound-based imaging, optical coherence tomography (OCT), and second-harmonic generation microscopy, all of which are non-invasive measures which can be used in a clinical setting to evaluate the quality of ECM deposition and assess the progression of wound healing.

#### 3.1.1 High frequency high resolution ultrasound imaging

Ultrasound allows for a holistic and non-invasive evaluation of a wound. This technique can analyze anatomy, hemodynamics, elastography, and volume of a wound while identifying variations in tissue type within the wound and quantifying morphological features. Moreover, current non-invasive techniques (thermography, macrophotography, laser speckle, perfusion mapping, and laser doppler flowmetry) only allow for superficial analysis of a wound. Ultrasound, on the other hand, allows for visualization of deeper structures, which greatly improves the ability to characterize full thickness wounds (Gnyawali et al., 2020). Clinical studies are ongoing which aim to characterize appropriately healing tissue parameters with high frequency ultrasound; tissues such as fibrous granulation tissue, cellular infiltrates, immature granulation tissue, and neoepidermis can be identified based upon their echolucency (Mohafez et al., 2018). Further advances in high resolution and microscopic ultrasound are allowing for precise characterization of ECM components and their orientation, which are promising techniques that may be applied to the monitoring of wound healing in the future (Morokov et al., 2019; Gnyawali et al., 2020). Gnyawali et al. (2020) have developed a novel non-invasive method of ultrasound imaging for repeated measure of wound tissue morphometry, biomechanics and hemodynamics under fetal regenerative, adult physiologic, and adult pathologic (diabetic wound) conditions using murine models. Their model uses high frequency, high resolution ultrasonography, coupled with doppler flow imaging to obtain hemodynamic properties of the blood flow in the artery supplying the wound-site and measurements of tissue cellularity and elastic strain for visualization of inflammation using Vevo strain software. They were able to characterize stark differences in elasticity, blood supply, and arterial pulse pressure that distinguish regenerative vs. fibrotic wound healing patterns. They further showed changes in the elasticity of wound-edge tissue of diabetic wounds, where the severe strain acquired during the early inflammatory phase persisted with a slower recovery of elasticity in the diabetic cohort as compared to that of the non-diabetic group. This imaging platform is versatile and clinically relevant for real-time analyses of wound healing and allows for multiple interrogations of the wound without disrupting the healing process, providing insight into the mechanical and functional aspects of the wound healing continuum, that can be readily applied to monitor wound healing in patients (Gnyawali et al., 2020).

In addition, high-frequency spectral ultrasound (SUSI) is an emerging technology that could provide a safe, portable, non-invasive diagnostic tool to detect ECM structure and fibrosis of various wounds including deep wounds through the fat, muscle, and bone along with dermis, and to the *epidermis*. SUSI, unlike conventional ultrasound imaging, takes advantage of a wide spectrum of signals based on radio-frequency backscattered signals to detect characteristic parameters independent of the function of the machine or the skill of the operator. SUSI allows for an objective and quantitative characterization of tissue. These spectral characteristics can be analyzed to identify specific tissue types through factors other than morphology (Ranganathan et al., 2018). In recent years, this technology has been used to successfully identify metastatic infiltration of biomaterials by cancer cells. This demonstration of cell specific identification is promising, as it demonstrates the capability of the technology to distinguish tissues at the cellular level and help to better characterize a wound’s healing status as described at the cellular, not gross morphological level (Bushnell et al., 2020).

#### 3.1.2 Elastography

Elastography is an ultrasound-based, non-invasive imaging modality that aids in the assessment of scaffold mechanical properties, such as volume, stiffness, and density (Sigrist et al., 2017). Recently, estimates of ECM stiffness and other stromal components using this technique has been used as a biomarker in assessing tumor microenvironments, as increased estimated tumor stiffness correlates to increased collagen density and fibroblast-rich environments (Riegler et al., 2018). Moving beyond the tumor environment, elastography was next used to evaluate tendon healing following reconstruction, providing non-invasive means to monitor successful healing and mechanical tissue properties (Gulledge et al., 2019; Frankewycz et al., 2020). More recently, shear wave elastography has been applied to objectively measuring human scar stiffness, where it reliably measured the stiffness of burn scars in a non-invasive manner (DeJong et al., 2020). This study further demonstrated that novice ultrasonographers became just as reliable as experienced ultrasonographers following a short training session in, further supporting elastography’s possible role in a bedside clinical environment of wound evaluation. Elastography is limited in its use, however, as its spatial *resolution* is large and is unable to resolve microscopic details necessary for evaluation of collagen microstructures.

#### 3.1.3 Optical coherence tomography

Optical coherence tomography scatters low-energy, high-wavelength light within a specimen to construct an image of the tissue structure and offers high-resolution, multi-sectional imaging. This modality has been widely used, in both experimental and clinical settings, to visualize complex microarchitecture assemblies at the micrometer level and has been extensively reviewed (Cannon et al., 2022; Groux et al., 2022; Ji et al., 2022; Sanchez et al., 2022). With regards to ECM characterization, it allows for rapid quantification of the amount of ECM that has been produced, as well as identification of factors that affect production rate such as cell proliferation and assessment of collagen microchannels within the ECM (Bagnaninchi et al., 2007). Recent advances have combined OCT with diffusion-sensitive technology using gold nanorods (DS-OCT) to increase the resolution from micrometers to nanometers, in order to appropriately distinguish collagen-based heterogeneity within the matrix (Blackmon et al., 2016).

**FIGURE 3:**
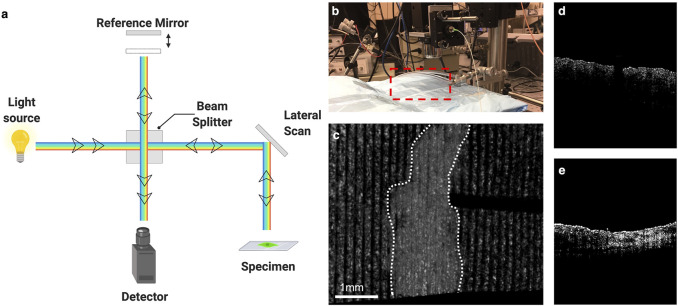
Optical coherence tomography and elastography. **(A)** Schematic of optical coherence tomography. **(B)** Set up for Optical Coherence Elastography. Red box designates location of sample/subject of interest. **(C)** Elastographic map of mouse skin with scar denoted within white dotted lines. **(D)** Cross section of normal skin using OCE. **(E)** Cross section of scar using OCE. Illustration generated using Biorender.com. OCE images courtesy of Dr. Kirill Larin Lab at the University of Houston.

Within the last few years, a new technique has emerged that combines the spectral domain of OCT with the ultrasound transducer of elastography, called ultrasound and phase sensitive optical coherence elastography (US-OCE). An acoustic radiation frequency from the ultrasound wave perturbs the surface of the specimen, resulting in a level of displacement (Figure 3). This displacement is then measured by the OCT system. Though seemingly simple, this technique combines the strengths of both independent methods. US-OCE has greater spatial resolution and is capable of quantifying the deformations in the specimen, providing information on the properties of the material as well (Wu et al., 2015; Liu et al., 2016; Nair et al., 2019). Though it has mostly been tested in lenses and small bowel tissue, OCT was recently trialed in a human study of split thickness skin grafting to monitor vascular structures and integration of the graft into the tissue (Deegan et al., 2021). This technique is well suited to study ECM stiffness and structure in wound healing.

#### 3.1.4 Single-photon, multi-photon, and second harmonic generation imaging

Optical imaging techniques such as single and multi-photon microscopy are uniquely suited to visualize and quantify ECM structure with minimal invasion over long periods of time, thereby providing exquisite detail of the inherently dynamic wound ECM remodeling from time of injury to weeks or months of remodeling. As the microstructure of the collagen is a primary determinate of the mechanical properties of the scaffold, the bulk optical properties from imaging could be used to predict bulk mechanical properties of the ECM.

Fluorescence-based single-photon and multi-photon microscopy with specifically targeted probes capable of the detection of ECM components can be used to assess the ECM composition. Recent studies have utilized collagen-mimetic peptides (CMPs), which are synthetic chains of amino acids and replicate the strands in natural collagen fibers, to detect damaged collagen (Dones et al., 2019; Ellison et al., 2020). However, the availability of specifically targeted probes with clinically approved fluorescence agents, such as Indocyanine green (ICG), and instrumentation is still limited. As well, molecular imaging enables non-invasive visualization of cellular and subcellular processes that may allow early detection, quantification, staging, and phenotyping of fibrosis. The role of molecular imaging probes for detecting fibrosis and fibrogenesis, the active formation of new fibrous tissue, and their application to models of fibrosis across organ systems and fibrotic processes has been reviewed elsewhere (Montesi et al., 2019). The availability of probes for the detection of ECM molecules, including collagens type I and III, oxidized collagen, fibrin and elastin, as well as for detection of neutrophil degranulation and vascular leak makes this imaging methodology much appealing to study cutaneous wound healing progression. Furthermore, these PET/SPECT probes require very low mass doses and have the properties of rapid clearance from the blood pool with low uptake in other organs making it highly translational.

Multiphoton microscopy is capable of high resolution, three-dimensional imaging of the ECM to a depth on the order of 1 mm using femtosecond pulses of near-infrared (NIR) laser light. Fibrillar collagen responds to near-infrared laser light with both second harmonic generation (SHG) and two-photon excited fluorescence (TPF). SHG microscopy uses the second-order, nonlinear optical response to visualize tissue microarchitecture without tagging or destruction of the specimen (Guo et al., 1997). SHG occurs when two incident photons interact with the non-centrosymmetric triple-helical structure of collagen and combine to form a single emitted photon of exactly half the wavelength or twice the energy, offering a label-free measure of intact collagen with high spatial resolution. During the remodeling phase, collagen type III is replaced with collagen type I, thus changing the overall collagen architecture. The changes in collagen fiber layering and deposition in the creation of the new ECM can be directly visualized and tracked with this method. Recent studies have detailed the imaging of collagen fibers in the skin, the lung, and the retina (Mostaco-Guidolin et al., 2017). Tanaka et al. (2013) performed SHG imaging on *in vivo* samples to observe changes in dermal collagen fibers in living rat burn models. The strength of this imaging technique relies on its use of the collagen dipole to discern orientation, since, in wound healing, collagen fibrils tend to present in a non-linear orientation. Combining SHG that detects intact collagen only, with fluorescence-based single-photon detection of denatured or fibrillar collagen with probes, will provide a clear demarcation of normal skin, wound boundary, and fibrotic remodeling. A drawback of SHG, however, is that it is limited to providing a two-dimensional representation of the tissue of interest. Furthermore, this technique is expensive, with bulky equipment that utilizes non-eye-safe class IV lasers, thereby limiting its clinical application. In contrast, 3D second harmonic generation tomography (SHT) rotates the specimen to collect images from all three planes to create a 3D image (Campbell et al., 2017), which can help characterize the intricate collagen assemblies within the ECM.

## 4 Computational modeling of the ECM

With massive innovations in sensing and imaging technology, multitudes of multimodality data will be collected which can be used to instantaneously understand the progression of a healing wound and predict the final healing outcome. However, the amount of data to be processed will be too burdensome or impossible for simple human interpretation, thus computational models of the ECM and the healing wound must be developed. Using these models will facilitate wound parameter extraction and processing, linking the qualities of the healing wound which will predict successful or failed wound healing outcome, allowing the wound care provider to intervene in a timely fashion to improve the chances of optimal wound healing.

### 4.1 Modeling in the different stages of wound healing

Much work has been done to model the proliferative phase of the wound healing process, as this phase represents the period of fibroblast proliferation and ECM deposition which closes the wound. These models are two-to-three variable models that attempt to simulate proliferation of cells as functions of tissue oxygen tension, capillary and fibroblast density, and/or mechanical factors including cell traction and ECM deformation. Initially, inputs to these numerical models included the presence of endothelial cells, macrophage-released chemokines, and new blood vessels (Pettet et al., 1996). Later models evolved to include vascular networks and interactions between independent and dependent factors, executed by modeling angiogenesis as a function of VEGF or tissue oxygen tension (Dor et al., 2003; Schugart et al., 2008). Most recent models included several ECM components such as ECM deformation and cellular traction forces, as well as other wound conditions like growth factor, oxygen concentrations, and the presence of new blood vessels and fibroblasts (Valero et al., 2014). The purpose of these models is to mathematically simulate optimal ECM formation in wound healing, which then allows researchers to define pathologic states of wound healing such as in the case of bacterial contamination and inadequate perfusion.

Mathematically modeling the remodeling phase is complex, as this phase is characterized by the interaction of variety of factors. Schluter et al. (2012) proposed that, within this phase, ECM fibers represent one of the most important factors guiding cellular migration to the wound. By estimating cell speed and size, total traction forces on the ECM, and ECM rearrangement due to these traction forces they found that 1) matrix stiffness and density leads to decreased cell movement (increased persistence of the cells within the matrix), 2) more orderly matrix structure leads to increased persistence, and finally, 3) in general, under wound healing conditions, cells tend to follow one another and matrix stiffness influences this behavior positively (Schluter et al., 2012). Using models such as these allow researchers to optimize the ideal wound stiffness for cellular reparative function and then develop techniques to measure stiffness in non-invasive ways.

### 4.2 Holistic wound healing evaluation with data-driven models

Integrating the three distinct healing phases, inflammation, proliferation, and remodeling, into a coherent system has been a central challenge of devising computational models of ECM and wound state dynamics. Numerical solutions that couple wound closure due to cell migration and angiogenesis have been explored using finite element and finite difference methods to solve the diffusion-reaction equations that define the physiology of the system and the hyperbolic equations governing the interface (the skin).

More advanced approaches have focused on introducing disease or injury states to the computational model used to describe ECM and tissue dynamics. Surgical tension wound states, tissue necrosis, and tissue ischemia have all been explored as additional conditions in which models have been compared to experimental findings with predictive success (Xue et al., 2009; Tepole et al., 2014; Valero et al., 2014). In addition, these models with pathologic states that complicate wound healing also serve as platforms to explore novel therapeutic factors to reverse and regenerate tissues.

All the models described thus far have been non-linear, partial differential equations systems with finite element methods solutions. While models such as these can use single parameter inputs to predict an expected outcome, they are unable to take conglomerate multimodality data and extract a computed outcome. In the data science realm, models that use machine learning may serve to help predict wound outcomes and guide decision making, but unlike differential equations systems, they can learn and adapt from the data they are given over time. Due to the high dimensionality of imaging and sensing data, most deep learning models have focused on wound stratification in images, but recently, models have been developed on risk stratification and healing prediction of wounds based on a large database of images and patient demographics (Wang et al., 2015; Li et al., 2018; Moccia et al., 2019). These two types of models are summarized in Table 1. Future models may be able to combine the two techniques, to create a learning framework that uses ECM composition and structure, as well as known signaling pathways of patient-specific wounds to predict wound healing outcomes. This can become a powerful tool to help physicians guide their decision making.

**TABLE 1:** Advantages and disadvantages of partial differential equation systems and deep learning systems.

	Non-linear partial differential equations system	Deep neural network/Deep learning system
Advantages	Can see and control input variables	Hierarchical feature learning ability transforming high-dimensional data into low-dimensional latent features
	Ability to model highly complex systems as a function of specific input variables	Ability of integrating multimodal data
		Ability to handle noisy data
Disadvantages	Labor and computationally intensive	Computationally expensive
	Difficult to change model given new data	Less interpretability
Selected Readings	Alber et al. (2019)	Wang and Gao (2019)
	Benhammouda and Vazquez-Leal (2014)	Ching et al. (2018)

### 4.3 Machine intelligence for wound healing approaches

With the many and varied new methods under development for collecting extremely large amounts of data surrounding the status of the ECM and a wound’s progression through the phases of the healing process, there is an unprecedented need for high dimensional data modeling to analyze the information and allow wound care providers to make informed decisions regarding treatment without significant delays. Thus, it is imperative to process high dimensional data, which has been acquired with a high sampling rate, into real-time feedback to enhance the system’s performance, and guide physician interventions based upon predicted wound healing outcome. To do this, various advancements in data-driven approaches with machine learning have been developed and utilized. The future may hold an adaptive “closing of the loop”, allowing an artificial controller to read the outputs of the computational algorithms from the multi-modal sensing to accurately assess the wound state in real-time, then trigger smart delivery systems to release therapeutics and drugs, without provider decision making.

#### 4.3.1 Machine learning

**FIGURE 4:**
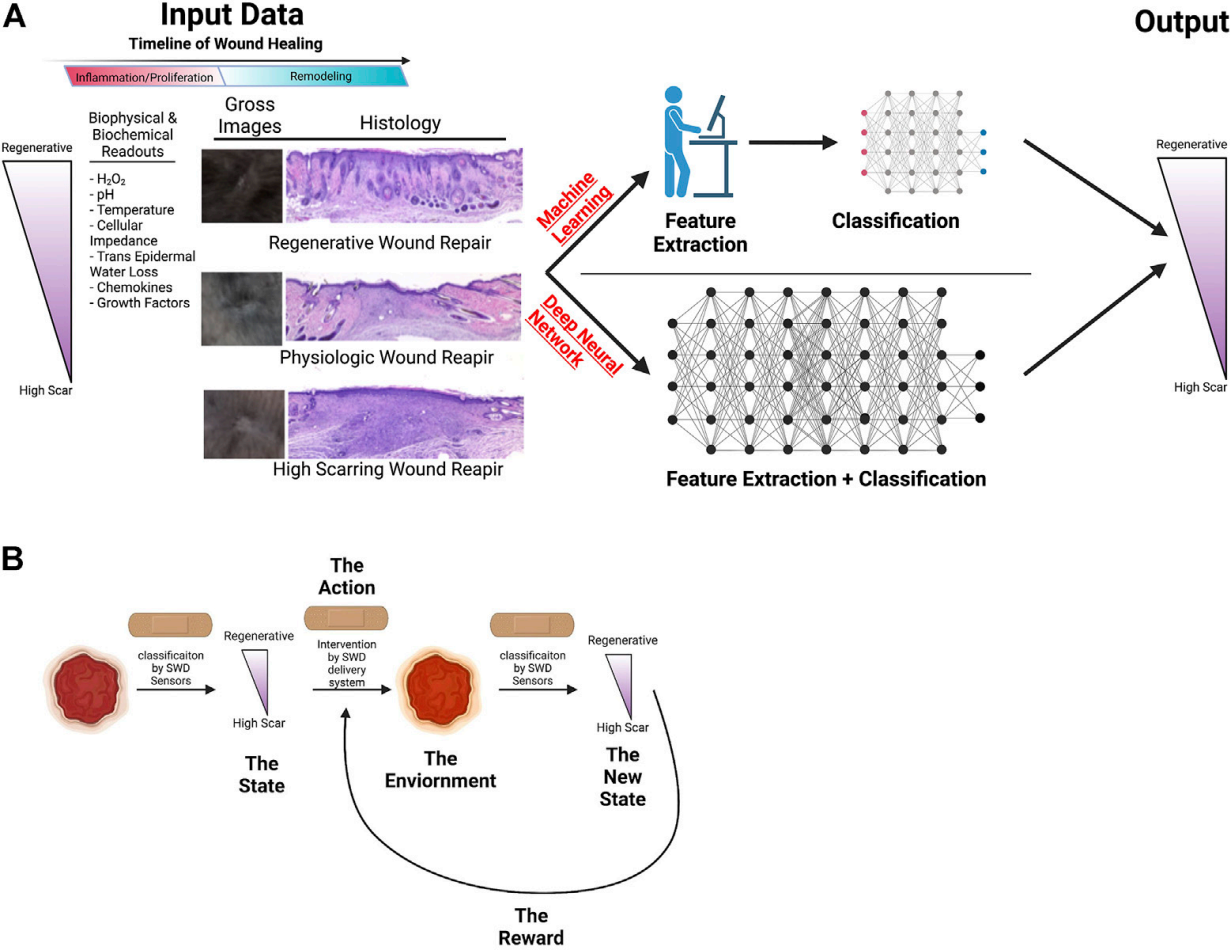
Illustration of potential machine learning applications to wound healing. **(A)** Schematic of machine learning vs. deep neural network use to improve wound healing outcomes. **(B)** Machine learning to modulate interventions to improve wound healing. Illustration created using Biorender.com.

Machine learning models have been used in the wound healing space to judge the state of wound healing based on wound measurements, gross images, and spatial frequency-domain imaging by a number of groups (Papazoglou et al., 2010; Rowland et al., 2019; Berezo et al., 2021; Squiers et al., 2021). Using machine learning algorithms trained on data gained in both human and animal experiments, models can be developed to determine the state of a wound based on the analysis of hand-picked, meaningful, and clinically relevant biophysical, biochemical, and histologic parameters, using linear regression models mapping sensor measurements to linearized approximation of the wound state. This kind of feature extraction has previously been demonstrated through the segmentation of clinical images and analysis of distinct and explicit biomarkers such as texture, shape, and color in order to diagnose and evaluate the clinical state of wounds in a variety of clinical pathologies (Cui et al., 2019; Kassem et al., 2021; Oukil et al., 2021). For example, the IDx-DR 2.0 algorithm has been utilized to detect diabetic retinopathy through feature extraction and analysis of clinical images of the retina (Abramoff et al., 2016; Obeid et al., 2019). Moreover, a classical machine learning pipeline utilizing NIS-Elements AR to analyze re-epithelialization of burn wounds has been validated through intraclass correlation to clinician analyzed images (Bloemen et al., 2012). This demonstrates the viability of machine learning algorithms to successfully and meaningfully predict wound outcomes as compared to the current standard of care. However, classic machine learning requires an expert, such as a physician, to curate the data processed by the machine, in essence informing the machine as to what input to “focus” on (feature extraction) in developing predictive model. This method of data analysis allows for precise and quantifiable feature extraction guided by experts that can be easily cross-validated to improve accuracy. However, the need for human influence in machine learning can allow unrecognized or uncharacterized patterns in the data to go overlooked. This weakness can be overcome by utilizing deep neural network modeling with reinforcement learning (Figure 4).

#### 4.3.2 Deep neural network modeling (deep learning)

Improving upon explicable models to predict wound healing outcomes, deep neural networks (DNN) represent a potential powerful tool for evaluation of healing wounds, as it allows for analysis of wounds without explicit feature extraction, allowing for previously unrecognized patterns to be analyzed and quantified. As the data collected by the above methods becomes greater in complexity and quantity, using machine learning becomes burdensome for correction and improving the trained models. DNNs differ from machine learning in that they are capable of hierarchical feature extraction which eliminates burdensome and costly feature engineering, thus allowing for simultaneous evaluation of greater magnitudes of data throughput. Over the past several years, DNNs have proved to be powerful tools for a wide range of modeling and prediction tasks, and have been shown to be effective in the diagnosis and prognostication of a variety of different pathologies (Janowczyk and Madabhushi, 2016; Cui et al., 2019; Chan et al., 2020; Morid et al., 2021; Yang et al., 2021). For example, like the explicable model, a DNN would take biophysical and biochemical inputs from the wound and output a prognosis for the wound. A novel application of neural networking to wound healing is processing simple photographs of wounds into a segmentation mask from which the wound area can be extracted (Cui et al., 2019), which aids in negating the significant burden of subjective inaccuracy of the human-performed measurements. This directly impacts patient care as the improvement in diabetic wound size in the first 4-weeks of healing is predictive of successful ultimate wound healing (Sheehan et al., 2006; Lavery et al., 2008; Sprigle et al., 2012).

Utilizing reinforcement learning makes sequential decision making possible. Reinforcement learning is the goal-directed learning exercised by biologic systems driven by achieving rewards while avoiding punishment (Wang et al., 2021). Machine Learning and DNN models are not suitable for sequential decision making in the changing environment. The power of reinforcement learning not only lies on the fact that the quality of each action is not measured by immediate reward, but also that it can discover the sequence of decisions (optimal policy) that maximizes the reward in unseen environment.

Finally, as with any image analysis, it is necessary to consider image quality and standardization. In order to overcome the inherent variation in image quality, lighting, and other factors, it has been shown that a composite approach in which images are pre-processed and segmented allows utilization of DL without the requirement of extensive training as previously required (Li et al., 2018). As well, this issue can be minimized by creating a standard procedure for image acquisition, with successful implementation in place by the Diabetic Foot Consortium through their eKare database of patient DFU images (Mamone et al., 2020). While in the past DNN have been considered as an undecipherable “black box”, recent studies have shown that it is possible to visualize explanations of features extracted by convolutional neural networks (CNNs) by utilizing the flowing gradient to final CNN layer to create a localization map highlighting important regions in the image for predicting the concept (Selvaraju et al., 2019).

Moreover, there are a variety of commercially available architecture CNNs that have been validated in a variety of clinical settings, ranging from skin wound healing to the detection of lung damage in SARS-CoV2 patients from lung images (Ohura et al., 2019; Yang et al., 2021). With the availability of such options, it is necessary to consider the many upshots and pitfalls for each, and develop image analysis pipelines that can reliably and reproducibility used for modeling.

#### 4.3.3 Closing the loop (artificial intelligence)

Current advances in computational analysis in wound healing aim to culminate in an entirely hands off implementation and utilization of the predictive data given by the machine learning and DNN models to improve wound healing with adaptive treatment regimen. This can be accomplished by designing and developing smart dressings that can sense various biophysical and biochemical parameters and deploy interventional therapeutics. The outputs from the models can feed back into an onboard controller that, using well understood and highly interpretable classical linear control systems, will enable tuning of interventions to correct for deviations from the ideal progression through the wound healing phases along a number of variables (Kailath, 1980). This process can be further fine-tuned by implementing reinforcement learning, where the algorithms detect the effects of interventions on the wound state and determine if the intervention had the desired outcome, reinforcing said intervention if it worked, and eliminating it if it has an adverse effect (Wang et al., 2021). By comparing outcomes of a given intervention to the expected outcome of the ideal intervention and minimizing the gap between these two variables, the smart dressings will be able to adapt to unique wounds, patients, and contexts. This technology exists already in the form of Q-learning and policy gradients, although it has yet to be utilized in a smart wound dressing device format (Watkins and Dayan, 1992; Peters and Schaal, 2008).

## 5 Conclusion

The ECM in a healing wound is a critical and active regulator of cell behavior. Its composition and structure are key modulators in preventing aberrant scarring and fibrosis. Novel physiologic biosensors, advanced imaging techniques, mathematical models, and *in vitro* and *in vivo* studies all contribute to our overall understanding of the changes that take place during the inflammatory and remodeling phases of wound healing which determine the structural and functional quality of ECM through the healing process. Translating these pre-clinical methods of non-invasive wound measurements and data processing, providers will be able to faithfully monitor, predict, and possibly intervene in the wound healing process to improve difficult-to-heal wounds.

## Abbreviations

DAMPs, damage associated molecular patterns; ECM, extracellular matrix; DR, dynamic reciprocity; EPS, extracellular polymeric substances; IM-MS, ion mobility mass spectrometry; H_2_O_2_, hydrogen peroxide; Pt-Black, Platinum-Black; ICGFA, indocyanine green fluorescence angiography; SUSI, high-frequency spectral ultrasound; OCT, optical coherence tomography; DS-OCT, diffusion-sensitive optical coherence tomography; US-OCE, ultrasound sensitive optical coherence elastography; SHG, second harmonic generation; CMPs, collagen-mimetic peptides; ICG, indocyanine green; NIR, near-infrared; TPF, two-photon excited fluorescence; SHT, second harmonic generation tomography; DNN, deep neural networks; CNN, convolutional neural network.
